# Regional Specializations of the PAZ Proteomes Derived from Mouse Hippocampus, Olfactory Bulb and Cerebellum

**DOI:** 10.3390/proteomes3020074

**Published:** 2015-05-13

**Authors:** Jens Weingarten, Melanie Laßek, Benjamin F. Mueller, Marion Rohmer, Dominic Baeumlisberger, Benedikt Beckert, Jens Ade, Patricia Gogesch, Amparo Acker-Palmer, Michael Karas, Walter Volknandt

**Affiliations:** 1 Department Molecular and Cellular Neurobiology, Institute for Cell Biology and Neuroscience, Goethe University Frankfurt am Main, Max-von-Laue-Straße 13, 60438 Frankfurt am Main, Germany; E-Mails: Weingarten@bio.uni-frankfurt.de (J.W.); Lassek@bio.uni-frankfurt.de (M.L.); bbeckert@stud.uni-frankfurt.de (B.B.); ade@stud.uni-frankfurt.de (J.A.); Acker-palmer@bio.uni-frankfurt.de (A.A.-P.); 2 Institute of Pharmaceutical Chemistry, Goethe University Frankfurt am Main, Max-von-Laue-Straße 9; 60438 Frankfurt am Main, Germany; E-Mails: Benjamin.mueller@gmx.de (B.F.M.); rohmer@pharmchem.uni-frankfurt.de (M.R.); karas@pharmchem.uni-frankfurt.de (M.K.); 3 SunChrom Wissenschaftliche Geräte GmbH, Industriestraße 27, 61381 Friedrichsdorf, Germany; E-Mail: dbaeumlisberger@sunchrom.de; 4 Paul-Ehrlich-Institute, German Federal Institute for Drugs and Medicinal Products, Paul-Ehrlich-Straße 51-59, 63225 Langen, Germany; E-Mail: patricia.gogesch@pei.de

**Keywords:** cerebellum, hippocampus, olfactory bulb, proteome, presynaptic active zone

## Abstract

Neurotransmitter release as well as structural and functional dynamics at the presynaptic active zone (PAZ) comprising synaptic vesicles attached to the presynaptic plasma membrane are mediated and controlled by its proteinaceous components. Here we describe a novel experimental design to immunopurify the native PAZ-complex from individual mouse brain regions such as olfactory bulb, hippocampus, and cerebellum with high purity that is essential for comparing their proteome composition. Interestingly, quantitative immunodetection demonstrates significant differences in the abundance of prominent calcium-dependent PAZ constituents. Furthermore, we characterized the proteomes of the immunoisolated PAZ derived from the three brain regions by mass spectrometry. The proteomes of the release sites from the respective regions exhibited remarkable differences in the abundance of a large variety of PAZ constituents involved in various functional aspects of the release sites such as calcium homeostasis, synaptic plasticity and neurogenesis. On the one hand, our data support an identical core architecture of the PAZ for all brain regions and, on the other hand, demonstrate that the proteinaceous composition of their presynaptic active zones vary, suggesting that changes in abundance of individual proteins strengthen the ability of the release sites to adapt to specific functional requirements.

## 1. Introduction

Communication between neurons involves chemical signaling via synaptic contacts consisting of the presynaptic neurotransmitter release site, the synaptic cleft, and the receptor-loaded postsynaptic site. Synaptic signaling is governed by the concerted action of a large variety of proteins. Due to the refinement of mass spectrometric methods proteomic studies of murine brain-derived synaptosomes, synaptic vesicles, postsynaptic densities, and presynaptic active zones (PAZs) recently provided increasing information on the proteomic composition of chemical synapses and their subcompartments (reviewed in [1]). These synaptic proteomes provide the basis for studying the interactome of the molecular constituents identified (reviewed in [2]). In contrast to the complex dynamics of postsynaptic densities [3,4], the dynamic composition of the PAZs is less well characterized, but now arouses increasing interest as a molecular platform of presynaptic plasticity (reviewed in [1]). The structural and functional dynamics at synaptic contacts in the adult CNS are reflected by presynaptic rearrangements of the proteinaceous inventory [5]. The detailed understanding of regional specializations requires the analysis of the proteomes of the presynaptic active zone (PAZ) from specific brain regions. Based on our experimental expertise for immopurifying the PAZ from rat [6,7] and mouse brain [5,8], we developed a novel experimental approach for the purification of subregion-specific PAZ proteomes. By combining stringent subcellular fractionation with subsequent immunopurification, we obtain a highly purified native PAZ proteome. This is now exploited for comparing the proteinaceous composition of the PAZ from mouse olfactory bulb, hippocampus and cerebellum by quantitative immunodetection and mass spectrometry. All these brain regions are involved in synaptic plasticity, in memory formation and memory consolidation. We hypothesized that the presynaptic release sites of the neuronal subpopulations in these brain regions are equipped with proteomes adapted to their circuitry-specific functions.

For example, the olfactory bulb receives via the glomeruli odorant information from the olfactory epithelium. Willful and unconscious odorant signal processing takes place by an interplay of neuronal populations prior to odor processing in higher brain regions. The olfactory bulb is also the target region of migrating neuroblasts that originate in the neurogenic niche of the subventricular zone and potentially involved in olfaction plasticity [9,10,11]. The hippocampal formation receives its input via the perforant path to the dentate gyrus. The concerted action of the hippocampal neuronal populations contributes to long-term memory formation. The hippocampus harbors a neurogenic niche, the subgranular zone of the dentate gyrus. Newborn neuroblasts migrate into the granule cell layer of the dentate gyrus and differentiate into interneurons potentially involved in declarative memory formation and consolidation [12,13]. The cerebellum plays an essential role in motor control and may also be involved in cognitive functions such as attention and language. Its neuronal network is important for motor memory acquisition and storage of complex motion sequences [14,15].

Our proteomic analyses reveal region-specific differences in the proteinaceous components of the PAZ proteomes concerning, e.g., calcium homeostasis, synaptic plasticity and neurogenesis, implicating adaptions to specific functional demands. These data present novel insight into the PAZ proteome and provide a solid basis for further characterizing differences in the proteinaceous inventory of the release sites derived from distinct brain regions.

## 2. Experimental Section

### 2.1. Animals

Animal treatment was performed under veterinary supervision according to European Guidelines. Mouse strain C57BL/6N was purchased from Charles River (Sulzfeld, Germany). Mice of both sexes, 3 months of age, were kept under 12 h light and dark cycles with food and water *ad libitum*.

### 2.2. Antibodies

Antibodies were directed against amyloid precursor protein (rabbit monoclonal, 1:1000, Abcam, Cambridge, UK), calbindin (rabbit polyclonal, 1:2000, Millipore, Darmstadt, Germany), CaMKII (mouse monoclonal, 1:1000, Abcam, Cambridge, UK), NCAM (rabbit polyclonal, 1:1000, Abcam, Cambridge, UK), Na^+^/K^+^-ATPase (mouse monoclonal, 1:500, gift of D. M. Fambrough), SV2 (the clone CKK 10H4 producing the monoclonal anti-SV2 antibody, kindly donated by Regis B. Kelly, San Francisco, CA, USA; was cultured in-house), synaptotagmin-1 (rabbit polyclonal, 1:1000, Synaptic System), Dynabeads M-280 conjugated with monoclonal sheep anti-mouse IgGs (cat. No. 112.02D), purchased from Invitrogen, Darmstadt, Germany.

### 2.3. Subcellular Fractionation of the PAZ from Mouse Brain Regions

Olfactory bulb, hippocampus, and cerebellum were dissected from native mouse brain prior to subcellular fractionation. Synaptic vesicles were isolated from synaptosomes according to the protocol guidelines of Whittaker [16]. The protocol has previously been adapted to the fractionation of individual mouse brains [5] and now downscaled for individual mouse brain regions (downscaling II). The following modifications were applied: individual brain regions (olfactory bulb, hippocampus, and cerebellum) were homogenized each in 0.4 mL of preparation buffer (5 mM Tris-HCl, 320 mM sucrose, pH 7.4) containing the protease inhibitors antipain, leupeptin, chymostatin (2 µg/mL each), pepstatin (1 µg/mL) and benzamidine (1 mM). Unless otherwise mentioned, the material was kept at 4 °C during the entire preparation. The brain homogenate was centrifuged using a Beckman TLX Optima 120 and rotor TLA 120.2 by acceleration (mode 4) up to 2800 rpm for 2 min. The resulting pellet was discarded and the supernatant was further fractionated by discontinuous Percoll gradient centrifugation. The Percoll gradient was prepared by layering 1.0 mL supernatant solution onto three layers of 1.0 mL Percoll solution (3%, 10%, 23% (*v/v*) in preparation buffer). After centrifugation using TLA 100.4 rotor for 7 min at 35,000 gav, fractions containing synaptosomes were collected and diluted twofold in preparation buffer and centrifuged using TLA 100.4 rotor for 35 min at 50,000 gav. For hypoosmotic lysis of synaptosomes the resulting pellet was triturated in four volumes of lysis buffer (5 mM Tris-HCl, pH 7.4) at room temperature. The suspension was centrifuged using TLA 100.4 rotor for 60 min at 250,000 gav. The pellet was resuspended and homogenized in 300 µL sucrose buffer (10 mM HEPES-NaOH, 0.5 mM EGTA, 0.1 mM MgCl2, 200 mM sucrose, pH 7.4). This microsomal solution was layered onto 900 µL of a discontinuous sucrose gradient (0.3 M, 0.75 M, and 1.2 M; containing 10 mM HEPES, 0.5 mM EGTA, adjusted to pH 7.4) and centrifuged using WX Ultra 90 Sorvall (Thermo Scientific, Bremen, Germany) and TST 55.5 rotor for 2 h at 65,000 gav. Thirty-six fractions (35 µL each) were collected from top to bottom of the gradient. The pooled lower fractions (LF) 16 to 30 corresponding to sucrose concentrations of 0.5 to 1.1 M were further analyzed.

### 2.4. Immunopurification of the Presynaptic Active Zone via Docked Synaptic Vesicles

The immunopurification protocol for the presynaptic active zone (PAZ) via docked synaptic vesicles, as described recently [5,7], was modified for individual mouse brain regions. In brief, 100 µL magnetic beads pre-coupled with an anti-mouse monoclonal antibody were washed with Tris-buffered saline (TBS, pH 7.4) and incubated with TBS containing 1% glycine, 1% lysine and 0.5% saponin followed by three washing steps in TBS. Magnetic beads were then incubated for 1 h with the anti-SV2 antibody (3 µg of antibody per 10^7^ magnetic beads to gain representative SV2 population). Crosslinking of the antibodies was performed with 0.1% glutardialdehyde in TBS for 5 min and stopped by adding TBS containing 1% glycine and 1% lysine. Finally, the beads were incubated over night at 4 °C with the pooled lower sucrose gradient fractions (LF, 16–30). Beads containing the bound material were washed three times with TBS and incubated with ice-cold acidified acetone (acetone containing 125 mM HCl) for 30 min at 20 °C. Elution was performed with different elution agents for 30 min. For Western blot analysis, proteins were eluted with sample buffer containing 2% SDS. For MS analysis proteins were eluted with 25 mM ammonium bicarbonate (ambic). The elution of PAZ proteins is supported by applying short ultrasonic pulses.

### 2.5. Lactate Dehydrogenase (LDH)

Lactate dehydrogenase (LDH) activity was determined as described by Johnson [17]. In brief, mouse brain homogenate was subjected to discontinuous Percoll gradient centrifugation as described. After centrifugation for 7 min at 35,000 gav 26 fractions were collected (130 µL each) from top to the bottom of the gradient. Ten microliters of sample were added to the substrate solution containing 150 mM NaCl with 50 mM Tris/HCl adjusted to pH 7.4, and 2.0 mg sodium pyruvate and 3.5 mg NADH+ were added per 50 mL. The amount of free LDH was measured using a spectrophotometer (Colora SPC 300, Hitachi, Tokio; Japan). Subsequently, 50 µL of 10% Triton X-100 was added to determine the total LDH activity (Katal per mL). The activity of occluded LDH was obtained by subtracting free from total LDH activity.

### 2.6. Western Blotting

For quantification of protein contents, the BCA-assay kit (#23225; Pierce, Rockford, IL, USA) was used. Immunopurified material was eluted from the beads with 2% SDS, 62.5 mM Tris, pH 6.8 prior to protein determination. The BCA kit tolerates up to 5% SDS, and 2% SDS are recommended to eliminate interference by lipids. Subsequently proteins were dissolved with sample buffer containing 2% SDS, 62.5 mM Tris, pH 6.8, 10% glycerol, and 0.01% bromophenol blue. Equal amounts of protein (100 ng) were resolved on a 15% Tris-glycine SDS-PAGE [18] and transferred onto nitrocellulose membrane (GE Healthcare, Solingen, Germany) using semi-dry blotting techniques (BioRad, Munich, Germany). Membranes were blocked with 5% skimmed milk powder in PBS/T (123 mM NaCl, 7.4 mM Na_2_HPO_4_, 4.3 mM KH_2_PO_4_, 0.1% Tween20) for 1 h. Incubation with the respective primary antibody was performed over night at 4 °C, followed by a second blocking step with 5% skimmed milk powder (five times, 10 min each), subsequent incubation with the respective HRP-conjugated secondary antibody (GE Healthcare) and a final washing step in PBS/T (five times, 10 min each).

### 2.7. Quantification and Statistics

Immunoblots were incubated with Western Lightning ECL substrate and visualized using ImageQuant LAS 4000 (both GE Healthcare). Quantification of immunosignals was performed with samples obtained under identical experimental conditions (*n* = 3–8) and run in one gel. Pixel intensities of non-saturated bands (±SEM, standard error of the mean) from the same blot were measured in voxels using ImageQuant TL software (version 8.1.0.0; GE Healthcare, 2011). Data were statistically processed employing unpaired Student’s *t*-test.

### 2.8. Mass Spectrometry—LC-MS/MS Analysis of Individual Brain Regions (B, H, C)

The immunopurified presynaptic active zone derived from mouse hippocampus, olfactory bulb and cerebellum was subjected to enzymatic digestion using the well-established serine protease trypsin. The amount of trypsin (Proteomics Grade, Sigma Aldrich, St. Louis, MO, USA) was adjusted to an enzyme-to-substrate ratio of 1:50 for each sample according to the protein concentrations determined by BCA Protein Assay (Pierce, Thermo Scientific, Waltham, MA, USA). The digestion was performed at 37 °C for 18 h and stopped by adding 3 µL of formic acid (FA). Samples were dried down and were solubilized in solvent A (5% MeCN, 0.1% FA) to obtain a final concentration of 1 µg peptide mixture per µL.

Chromatographic separation of peptides was performed using an EASY nLC II system (Thermo Scientific, Bremen, Germany). Both precolumns as well as analytical columns were filled in-house with XBridge BEH C18 material (3.5 µm, 130 Å, Waters, Eschborn, Germany) and an optimized gradient was applied with increasing amounts of solvent B (95% MeCN, 0.1% FA) during 130 min at 300 nL/min.

Mass spectrometric measurements were performed online using a micrOTOF-Q II ESI-Qq-TOF instrument (Bruker Daltonics, Bremen, Germany) equipped with a nano ESI source using the following parameters of a previously optimized acquisition method [19]: electrospray voltage 4500 V, end plate voltage 50 V, nebulizer gas pressure 0.4 bar, dry gas 4 L/min, dry gas temperature 180 °C, scan range 50–2000 *m/z*, scan rate 1.25 Hz. Nitrogen was used as collision gas with the flow rate set to 30%. Collision sweeping was set as active, with the collision RF changing from 800 Vpp to 200 Vpp. A maximum of seven precursors per MS spectrum was selected for MS/MS acquisition. Quadratic calibration was performed using a calibration tune mix for ESI measurements with extended mass range (Bruker Daltonics, Bremen, Germany).

Acquired spectra were post-processed using Compass Data Analysis (V4.0, Bruker Daltonics, Billerica, CA, USA) including deconvolution of spectra, detection of compounds and compilation of mascot generic format (MGF) files for database search. MS/MS searches were performed employing an in-house Mascot server (V2.4.1, MatrixScience Ltd., London, UK [20]) using the following parameters: 25 ppm peptide mass tolerance, 0.05 Da fragment mass tolerance, tryptic enzyme specificity, up to one allowed missed cleavage and ESI-QUAD-TOF as instrument setting. The database (SwissProt, released on 13 November, 2013) was restricted to murine proteins and false discovery rate (FDR) was estimated by a decoy search and set to be ≤1.5%. Protein identifications with two or more matched peptides were considered as significant.

## 3. Results

**Figure 1:**
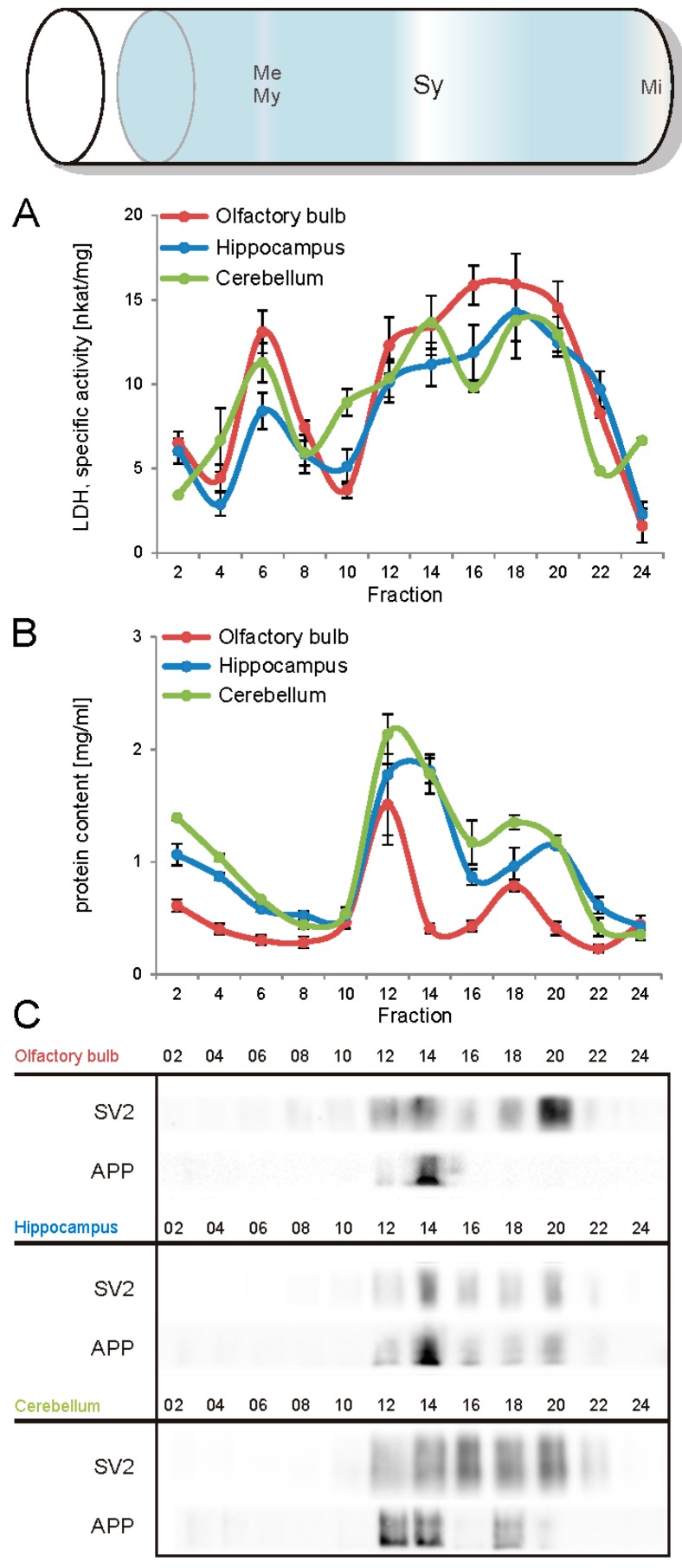
Analysis of the Percoll gradient for the enrichment of synaptosomes derived from olfactory bulb (red), hippocampus (blue), and cerebellum (green). **A**, specific activity of occluded LDH (nkat/mg of protein), **B**, protein content (mg/mL), **C**, immunodetection of SV2, a marker protein for synaptic vesicles and the amyloid precursor protein APP, a constituent of the presynaptic plasma membrane. The concomitant occurrence of occluded LDH (plateau) and synaptic vesicles indicates the sedimentation range of synaptosomes (fractions 12–20). APP, amyloid precursor protein; Me, membranes; Mi, mitochondria; My, myelin; SV2, synaptic vesicle protein 2, Sy, synaptosomes. Bar graphs are mean ± SEM, *n* = 3.

**Figure 2:**
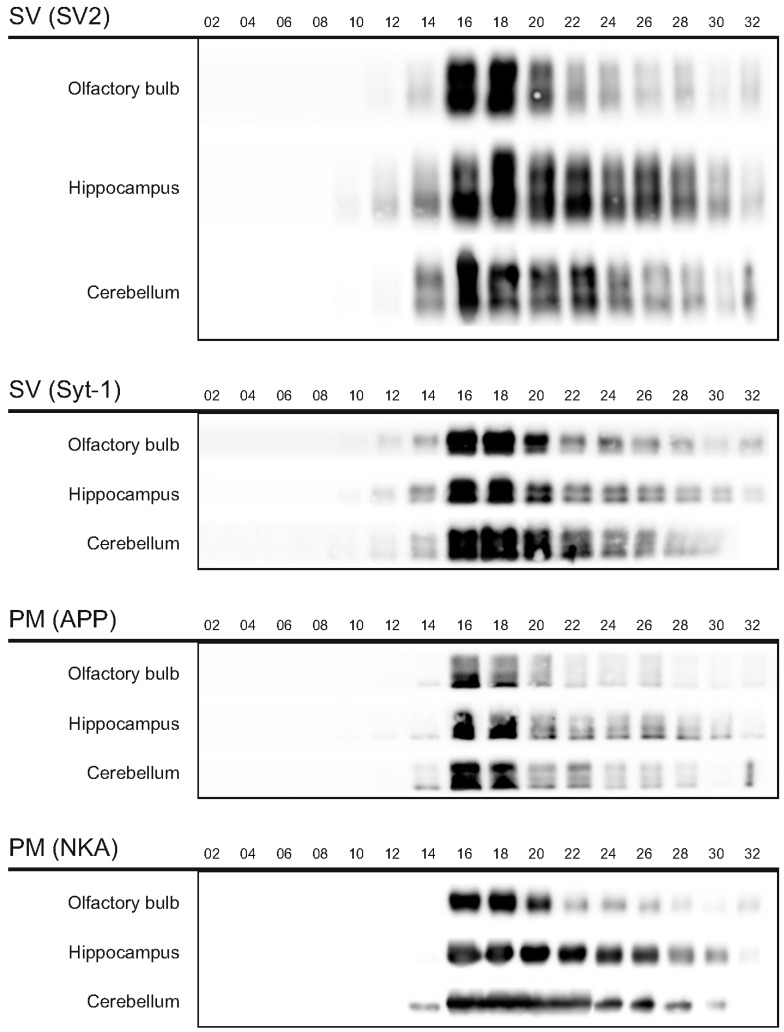
Migration of marker proteins upon sucrose gradient centrifugation. The synaptic vesicle proteins 2 (SV2, 80/86 kDa) and synaptotagmin-1 (Syt-1, 65 kDa), and the amyloid precursor protein (APP, 110 kDA), and Na^+^/K^+^-ATPase (NKA, 110 kDa) served as markers for the presynaptic active zone. PM, presynaptic plasma membrane; SV, synaptic vesicles. Six microliters of the respective fractions were applied per lane.

**Figure 3:**
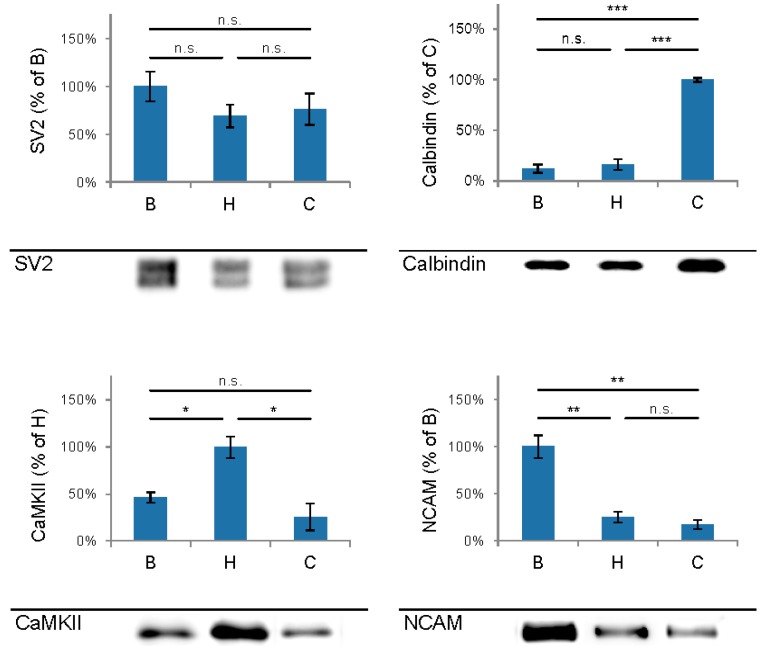
Comparison and quantification of the abundance of selected proteins of the immunopurified presynaptic active zone (PAZ) derived from olfactory bulb (B), hippocampus (H) and cerebellum (C). Bar graphs are mean ± SEM. * *p* ˂ 0.05; * *p* ˂ 0.01, * *p* ˂ 0.001, n.s. difference not significant. The highest value of the respective proteins was set to 100%. A representative Western blot is shown below each diagram. Equal amounts of protein were loaded per lane. SV2, *n* = 5; calbindin, *n* = 4; CaMKII, *n* = 3; NCAM, *n* = 4.

The first aim of this study was to immunopurify the proteome of the presynaptic active zone (PAZ) from defined mouse brain regions. Therefore we developed a new experimental protocol for subcellular fractionation and immunoisolation of the PAZ derived from olfactory bulb (B), hippocampus (H) and cerebellum (C), based in principle on the method for individual total mouse brain as previously described in detail [5]. For this purpose, we evaluated the key steps of subcellular fractionation for all three brain regions starting with the purification of synaptosomes via Percoll gradient centrifugation and followed by sucrose density gradient centrifugation of synaptic vesicles docked to the presynaptic active zone. Activity of lactate dehydrogenase (LDH) measurement was chosen as marker for the accumulation of metabolic active membrane-sealed compartments during Percoll gradient centrifugation. Sealed compartments containing synaptic vesicles were regarded as synaptosomes. Upon Percoll gradient centrifugation membrane occluded lactate dehydrogenase (LDH) revealed a peak in fraction 6 and a broad plateau ranging from fractions 12–20 (Figure 1A). The curve progression is highly comparable for the individual brain regions. Immunodetection of characteristic marker proteins of the PAZ, the ubiquitous synaptic vesicle protein SV2 and the presynaptic plasma membrane constituent amyloid precursor protein APP, revealed strong signals for SV2 and APP within fractions 12–20. The results demonstrate the simultaneous presence of membrane-occluded LDH and marker proteins, indicating an enrichment of metabolically intact nerve terminals referred to as synaptosomes. The synaptosome enriched fractions were pooled and subjected to hypoosmotic lysis prior to discontinuous sucrose gradient centrifugation and immunodetection. Marker proteins for synaptic vesicles (SV) SV2 and synaptotagmin-1, as well as the plasma membrane (PM) constituents APP and Na^+^/K^+^-ATPase (NKA) co-migrated to denser fractions (fractions 16–24), indicating the presence of synaptic vesicles attached to the presynaptic membrane via the SDS-resistant SNARE complex (Figure 2). These fractions were employed for immunoisolation of the PAZ using a monoclonal antibody directed against the 12 transmembrane span synaptic vesicle protein SV2. The immunopurified PAZ was subsequently analyzed by quantitative immunodetection to evaluate potential differences between PAZs derived from individual brain regions. The immunosignal for SV2—the target for immunopurification—revealed no significant differences between the PAZs derived from the three different brain regions (Figure 3) and yielded similar amounts of PAZ protein upon immunopurification. The fast calcium buffer protein calbindin was more abundant in the cerebellar PAZ as compared to the olfactory or hippocampal PAZ (*** *p* < 0.001). The calcium/calmodulin-dependent kinase CaMKII involved in presynaptic signaling and an important mediator of learning and memory was most abundant in the hippocampal PAZ (* *p* < 0.05), whereas the neuronal cell adhesion molecule NCAM (** *p* < 0.01) was most abundant in the olfactory PAZ (Figure 3).

**Figure 4:**
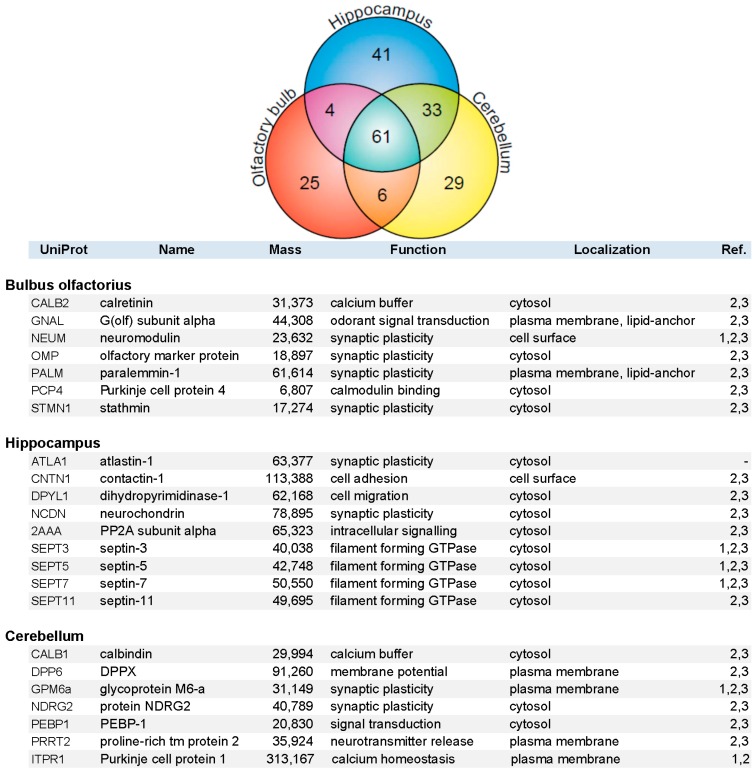
VENN diagram (upper graph) of proteins identified by mass spectrometry in the immunopurified PAZ derived from olfactory bulb (B, red), hippocampus (H, blue), and cerebellum (C, yellow). A total of 199 proteins were reproducibly identified with significance (FDR < 1.5%) with two or more peptides in a minimum of three independent experiments (B = 96, H = 139, and C = 129 proteins), yielding an overlap of 61 proteins. Whereas 25 (B), 41 (H), and 29 (C) individual proteins were identified in the respective PAZ only. A selection of individual PAZ constituents is listed below (1, [21]; 2, [6]; 3, [5]).

Constituents of the immunopurified PAZ from olfactory bulb, hippocampus, and cerebellum were further identified by mass spectrometry. We have identified 648 individual PAZ proteins in total (B: 359; H: 418; C: 424) including prominent constituents like: SV2, synaptotagmin-1, synaptophysin, SNAP25, synataxin-1, Munc-18 and Na^+^/K^+^-ATPase. The data highlighted here focus on the composition of select PAZ proteomes derived from different brain regions. Setting the threshold for proteins that could be reproducibly identified with a significance of FDR < 1.5% and with two or more peptides in a minimum of three independent experiments yielded 199 proteins (B = 96, H = 139, and C = 129 proteins), resulting in the high overlap of 61 proteins between the three brain regions (VENN diagram; Figure 4). This underpins the occurrence of common core constituents of PAZs and is in line with previous observations [5]. Furthermore, 25 (B), 41 (H), and 29 (C) individual proteins were exclusively identified within one of the respective brain regions. Core constituents of the PAZ abundant in all three brain regions included integral and associated synaptic vesicle proteins such as the SNARE-complex constituents VAMP2, SNAP25, syntaxin-1, munc18, the glycolytic machinery, signaling proteins such as 14-3-3 isoforms, CNPase, DRP-2, and subunits of CaMKII, plasma membrane-allocated proteins such as Thy-1 and NKA, the numerous cytoskeletal proteins involved in actin filament and microtubule dynamics, and spectrin (S1). A selection of proteins identified in only one of the respective PAZ and involved in calcium homeostasis and synaptic plasticity is listed in alphabetical order in Figure 4. With the exception of atlastin-1, these proteins have recently been assigned to the PAZ of the entire rat and mouse brain. These are involved in diverse functional aspects of the release sites such as calcium homeostasis (calretinin, calbindin, Purkinje cell protein 1), synaptic plasticity (neuromodulin, paralemmin-1, contactin-1, protein NDRG2), cellular dynamics (septins-3/5/7/11), and structural reorganization (stathmin, dihydropyrimidinase-1). Additional examples include the olfactory marker protein OMP that was exclusively identified in the PAZ derived from olfactory bulb, neurochondrin in the hippocampal PAZ, and calbindin in the cerebellar PAZ. This suggests that these proteins have an increased abundance in the respective PAZs.

## 4. Discussion

The presynaptic active zone represents a focal hot spot that is not only involved in the regulation of neurotransmitter release but also in multiple plastic structural and functional alterations underlying neural activity in the adult CNS [5]. The concerted action of a set of proteins present at the release site governs central functions in synaptic signaling (reviewed in [1,2]). Furthermore, constituents of the presynaptic active zone are targets of numerous potent neurotoxins and vulnerable to neurodegenerative diseases. Lassek and coworkers allocated the amyloid precursor family members APP, APLP1 and APLP2 to the release sites [8]. Here we present proteomic data from different brain regions that originate from heterogeneous populations of neurons. The highly purified native PAZ proteomes derived from olfactory bulb, hippocampus and cerebellum reveal a conserved common core composition, indicative of common functional and structural principles. Moreover, our data elucidate pronounced differences between brain regions in the abundance of PAZ constituents, implicating specific adaptions of the PAZ proteinaceous inventory to specific tasks in neural circuitry and plasticity. In agreement with previous data derived from entire rat [6,21] and mouse brain [5] mitochondria are common to all PAZ proteomes, whereas constituents of the postsynaptic density are virtually absent.

In the following we briefly highlight one selected protein with increased abundance in the PAZ of only one of the brain regions. In addition, selected proteins in the immunopurified PAZ from olfactory bulb, hippocampus and cerebellum are portrayed in the supplementary material (Text S2). Interestingly, many of these proteins are involved in adult neurogenesis and synaptic plasticity. Moreover, they have been implicated in learning and memory formation that is often impaired in neurodegenerative disorders.

The olfactory marker protein (OMP) was exclusively identified in the PAZ derived from olfactory bulb. OMP immunohistochemistry identifies olfactory receptor cell axons in the olfactory bulb [22]. OMP is present only in mature neurons [23]. Double labeling demonstrated that OMP and the microtubule-associated MAP2 are distributed in distinct regions within the glomerulus revealing the compartmental nature of subglomerular organization. The synaptic vesicle protein synaptophysin was found to strongly co-localize with OMP [24]. OMP knock-out pups fail to show preference between their biological mothers and unfamiliar lactating females [25].

The neuron specific neurochondrin was identified in the hippocampal PAZ. Prominent expression of neurochondrin in the adult brain was previously observed in hippocampus, amygdala, septum, and nucleus accumbens with moderate expression in the dorsal striatum [26,27]. Neurochondrin was originally discovered as a protein that induces neurite outgrowth [28]. A synaptosome fraction purified from mouse brain contained both neurochondrin and mGluR5 [27]. Neurochondrin knockout attenuated mGluR5-dependent stable changes in synaptic function—LTP and LTD—in the hippocampus [27]. Neurochondrin acts as a negative regulator of calcium/calmodulin-dependent protein kinase II phosphorylation and is essential for the spatial learning process [29]. Neurochondrin knockout led to a behavioral phenotype associated with an animal model for schizophrenia, as indexed by alterations both in sensomotoric gating and psychotomimetic-induced locomotor activity [27].

The EF-hand calcium binding protein calbindin was highly abundant in the immunopurified cerebellar PAZ. It binds calcium ions with high affinity [30] and is enriched in Purkinje cells [30,31]. Cells that displayed calbindin during brain development were also calbindin-positive in the adult animal. Positive cells represented 74% of the Purkinje cells from the cerebellar cortex, whereas less than 1% of the neurons in the frontal cortex were immunopositive for calbindin [32]. The adult expression pattern developed steadily in cerebellum [33] and in mature Purkinje cells with calbindin contributing about 15% of total cellular protein [34]. Selective deletion of calbindin from cerebellar Purkinje cells resulted in distinct cellular and behavioral alterations with permanent deficits of motor coordination and sensory processing [35,36].

In summary, the proteome of the immunopurified PAZ derived from olfactory bulb, hippocampus, and cerebellum revealed common core constituents that play a central role in presynaptic function. It is of note that the abundance of several of the protein constituents differed considerably between the respective brain regions, presumably reflecting region-specific functional adaptions of the presynaptic release site. This information helps to understand the impact of therapeutic drugs on their targets and to elucidate their subsequent effects on the PAZ proteome. In a similar way, the dynamics and functional diversity of the postsynaptic AMPA receptor proteomes was found to reflect context-specific modulation [4].

## 5. Conclusions

The identification of individual presynaptic active zone protein components is a prerequisite for further functional investigations and also provides a solid basis for evaluating their interaction. Our data suggest that the differences in the PAZ proteome reflect specific adaptions to regional neuronal circuitries and the functional and structural dynamics of their corresponding release sites. Moreover, our novel experimental setup opens avenues for studying presynaptic active zone proteomes in time and space under native conditions. The findings reported here may serve as a template also for studying the impact of brain region specific mutants on the presynaptic proteome and presynaptic physiology.

## Supplementary Materials

Supplementary File 1
