# Supplementary material for: Regional Specializations of the PAZ Proteomes Derived from Mouse Hippocampus, Olfactory Bulb and Cerebellum
Source: Proteomes. 2015 May 13;3(2):74–88. doi: 10.3390/proteomes3020074 (PMC5217373; doi:10.3390/proteomes3020074)
Supplement: Supplementary File 1 [file proteomes-03-00074-s001.zip › proteomes-03-00074-supplementary-S2.pdf]

Supplementary Material

1. Characteristics of Select Proteins Identified in the Immunopurified PAZ from Olfactory Bulb, Hippocampus and Cerebellum

The proteins (highlighted in bold) were selected from the data shown in the VENN diagram of Figure 4. To briefly portray individual proteins major characteristics reported in the literature are listed.

2. Constituents of the PAZ Derived from Olfactory Bulb

Purkinje cell protein 4, also called brain-specific polypeptide 19, is a neurospecific, small calmodulin binding protein, both calcium-free and calcium-bound, that regulates calmodulin-mediated signals [1]. Immunocytochemistry demonstrated that PCP4 is expressed predominantly in the cerebellum, especially Purkinje cells, piriform cortex, thalamic nuclei, caudate nucleus, putamen, cortex and hippocampus [2,3]. PEP-19 appears during the final stages of maturation of Purkinje cells. PCP4 promotes neurotransmitter release by activating calmodulin function and regulates calmodulin-dependent protein kinase II (CaMKII). Peptide antagonists of calmodulin (camstatins) based upon a conserved structural motif in PEP19, neurogranin, and neuromodulin block LTP and increases calcium extrusion in hippocampal CA1 neurons [4,5].

A proteome profile of the mature rat olfactory bulb identifies neuromodulin (NeuM, GAP-43, B-50) [6]. Immunocytochemistry reveals that neuromodulin is predominantly present in neuronal growth cones and preterminal axons [7]. It has been associated with neurite outgrowth [8,9], neurotransmitter release [10], and synaptic plasticity [11].

Stathmin-1 is a ubiquitous cytosolic phosphoprotein highly expressed in the developing nervous system and is maintained in some regions of the adult central nervous system. Immunohistochemistry revealed the presence in cells of the rostral migratory stream indicating an expression of stathmin in regions of the adult nervous system characterized by striking structural plasticity and cell renewal, suggesting that this protein could play a role in the differentiation of newly generated cell populations [12]. Stathmin-1 is related to the neuron-specific membrane-associated stathmin-2 (SCG10). Phosphorylation of both proteins negatively regulate the stability of microtubules in growth cones and act as cellular mediator of microtubule dynamics [13] and their upregulation play a role in axonal regeneration in the adulthood [14,15]. Whereas stathmin-2 is restricted to immature olfactory neurons stathmin-1 is also expressed in basal cells. Proteomic and immunochemical analysis postulated a role for stathmin in adult neurogenesis [16]. Stathmin interacts with tubulin, leading to microtubule destabilization [17]. In adults, stathmin is prominently expressed in neurogenesis pathways including the subgranular zone of dentate gyrus, subventricular zone, rostral migratory stream, and olfactory bulb [12,18].

Olfactory marker protein (OMP) immunohistochemistry identifies olfactory receptor cell axons in the olfactory bulb [19]. OMP is present only in mature neurons [20]. Double labeling demonstrated that OMP and the microtubule-associated MAP2 are distributed in distinct areas within the glomerulus revealing the compartmental nature of subglomerular organization. Axonal areas, identified by dense OMP-immunoreactivity, are found adjacent to areas devoid of OMP. The synaptic vesicle protein synaptophysin strongly co-localized with OMP [21]. OMP knock-out pups fail to show preference between their biological mothers and another unfamiliar lactating female [22].

Intracellular calcium, important in a variety of second messenger cascades, is regulated in part by 5 EF hand calcium-binding proteins such as calretinin, parvalbumin, calbindin, cal-modulin and neurocalcin in developing olfactory bulb [23,24]. These proteins were highly concentrated in the rat main olfactory bulb and were localized in distinct neuronal populations [25]. In adult olfactory bulb calretinin has been localized in mitral cells and interneurons [26]. The axons and especially the axon terminals of mitral cells are calretinin-immunoreactive [27]. The subventricular zone (SVZ) is known to be the major source of neural stem cells in the adult brain. In rodents and non-human primates, many neuroblasts generated in the SVZ migrate in chains along the rostral migratory stream to populate the olfactory bulb with new granular and periglomerular interneurons [28]. Most granular cells in the olfactory bulb are GABAergic and the vast majority of the granular cells that are generated during adulthood in rodents expressed a GABAergic phenotype [29]. GABA appears to be the major transmitter used by periglomerular neurons. A significant number of these neurons also contained the calcium-binding protein calretinin, whereas a smaller number expressed tyrosine hydroxylase, a reliable marker for dopaminergic neurons in the olfactory bulb [30]. Neurons containing parvalbumin, a calcium-binding protein often associated with GABA [31,32], were also detected in adult human olfactory bulb and their distribution overlapped that of the GABAergic neurons.

The lipid-anchored paralemmin is highly expressed in neuronal plasma membrane and has been implicated as a potent modulator of cellular cAMP signaling within the brain [33]. The olfactory neuron specific alpha subunit G(olf) of the trimeric G-proteins [34] involved in odorant sensing and signal transduction is located predominantly in the posterior subdivision of the accessory olfactory bulb [35,36].

3. Constituents of the PAZ Derived from Hippocampus

The dynamin superfamily member atlastin-1, an oligomeric GTPase, is enriched in pyramidal neurons in the cerebral cortex and hippocampus [37]. Atlastin-1 exists as an oligomer, most likely a tetramer, and is highly enriched in vesicular structures within axonal growth cones and variocosities as well as axonal branch points indicating a functional role of atlastin-1 during axonal development regarding axon formation and elongation [38].

Contactin-1 (F3) is a GPI-anchored neuronal cell surface adhesion molecule modulating interactions in developmental and regenerative processes. Contactin-1 is expressed at CA1 synapses, where it is selectively required for paired-pulse facilitation and NMDA receptor mediated long-term depression [39,40]. Contactin-1 assembles in a complex on the synaptic plasma membrane that regulates intercellular interactions necessary for specific modifications in synaptic stress. Contactin-1 plays a selective role in synaptic plasticity [39] and promotes adult hippocampal neurogenesis [41]. Overexpression of contactin-1 leads to increased CA1 long-term potentiation and improved spatial and object recognition memory [41].

Prominent expression of the neuron specific neurochondrin (Norbin – neurite-outgrowth re-lated rat brain protein) in adult brain was observed in hippocampus, amygdala, septum, and nucleus accumbens with moderate expression in the dorsal striatum [42,43]. Neurochondrin was originally discovered as a protein that induces neurite outgrowth [44]. A synaptosome fraction purified from mouse brain contained both neurochondrin and mGluR5 [43]. Neurochondrin knockout attenuated mGluR5-dependent stable changes in synaptic function—LTP and LTD—in the hippocampus [43]. Neurochondrin is a negative regulator of calcium/calmodulin-dependent protein kinase II phosphorylation and essential for the

spatial learning process [45]. Neurochondrin knockout led to a behavioral phenotype associated with an animal model for schizophrenia, as indexed by alterations both in sensomotoric gating and psychotomimetic-induced locomotor activity [43].

PP2A is a trimeric serine/threonine protein phosphatase composed of a scaffold subunit that is associated with a catalytic subunit and an additional, more variable, regulatory subunit that is expressed in a cell- and tissue-specific manner [46]. Long-term depression in the hippocampus was associated with an increase in the activity of PP1 and PP2A [47] that could be blocked by inhibitors of PP1 and PP2A [48]. These protein phosphatases could reduce extracellular signal-regulated kinase 2 (ERK2) in the adult hippocampus during LTD but not ERK1 [49]. PP2A is associated with Alzheimer neurofibrillary pathology. Phosphorylated tyrosine (Y307) in the catalytic subunit inactivated PP2A that was then unable to dephosphorylate tau [50]. The counter player the glycogen synthase kinase-3 inhibited PP2A and phosphorylated tau [50]. PP2A mRNA expression was quantitatively decreased in Alzheimer's disease hippocampus [51]. Over activation of PP2A was involved in lead-induced deficits in learning and memory [52].

The name septins was chosen to reflect the role of these proteins in separation of mother and daughter cells [53]. The septins 3/5/7 and septin 11 belong to a family of proteins with GTPase activity that form heterooligomeric filaments and ring-like structures that act as diffusion barriers and scaffolds.

Septins are involved in cytokinesis, positioning of the mitotic spindle, cellular morphology, vesicle trafficking, apoptosis, neurodegeneration, and neoplasia [54–56]. In mammals, 14 septin genes have been identified. Each septin gene is expressed in several spliced forms. Although most septins are highly expressed in the brain [57], only recently their role in neuronal function [58] and in neuropathological diseases such as Alzheimer`s disease [57], Parkison disease [59,60] and hereditary neuralgic amyotrophy [61] is beginning to be addressed. Septins can associate both with actin filaments and microtubules. Septins 3, 5, and 7 were localized in the presynaptic terminals, frequently associated with synaptic vesicles [57,62–65] and the presynaptic active zone [66,67]. Septin 3 was particularly abundant in mossy fiber nerve terminals in the hippocampus where it strongly co-localizes with synaptophysin and dynamin-1 [65]. In neurons, septin 11 forms heterooligomeric complexes with septin 5 and septin 7. Septins 3/5/7/11 exhibited an increase in expression from embryonic day 15 to postnatal day 70, and were abundantly expressed in axons and growth cones of developing hippocampal neurons, and were present in presynaptic terminals of mature synapses indicating a functional role within the presynapse. Knockdown of septin 5 or septin 1 in developing hippocampal neurons impaired axon growth [68].

Dihydropyrimidinase-related protein 2 has been identified as a constituent of the rat brain hippocampus proteome [69]. The function of dihydropyriminidase-related proteins 1 and 2 is not resolved yet, however both proteins have been implicated in axon growth and guidance [70]. Dihydropyrimidinase-2 regulated axon extensions [71] and neurogenesis [72]. Dihydropyrimidinase-related protein-2 demonstrated changes in protein abundance in a variety of neurological disorders such as Alzheimer`s disease [70,73], phenylketonuria mouse model [74], methamphetamine exposure [75], depression [71,72], kainite-induced pathology [76], and cellular nitrating conditions [77]. Dihydropyrimidinase-related protein 2 has been observed in an axogliasomal fraction [78] and has been linked to spatial memory formation [79]. The neurotrophin BDNF induced dihydropyrimidinase-related protein 2 expression changes in hippocampal neurons [80].

4. Constituents of the PAZ Derived from Cerebellum

Calbindin, named calbindin D-28k due to its vitamin D dependence and its apparent molecular mass, belongs to the large family of EF-hand calcium-binding proteins characterized by well conserved helix-loop-helix motives that bind calcium ions with high affinity [81]. Calbindin is enriched in Purkinje cerebellar neurons [81,82]. Cells which displayed calbindin during brain development were also calbindin positive in the adult animal. Positive cells represented 74% of the Purkinje cells from the cerebellar cortex, whereas less than 1% of the neurons in the frontal cortex were immunopositive [83]. Adult expression pattern developed steadily in cerebellum [84]. In mature Purkinje cells calbindin contributed about 15% of total cellular protein [85]. Selective deletion of calbindin from cerebellar Purkinje cells resulted in distinctly different cellular and behavioral alterations with permanent deficits of motor coordination and sensory processing [86]. Calbindin-deficient mice developed normally without upregulation of related calcium-binding proteins. However, when challenged in tests of movement coordination, severe ataxia became evident [87].

Dipeptidyl peptidase-like protein 6 (DPP6, also known as DPPX) is a type II membrane glycoprotein with a large extracellular C-terminal domain and a single transmembrane that revealed no enzyme activity domain [88,89]. DPP6 was expressed in cerebellar granule cells where it regulated resting membrane potential and input resistance [90] suggesting a role for DPP6 in sculpting the high frequency excitability of cerebellar granule cells [91]. DPP6 is an integral auxiliary subunit of the Kv4.2 potassium ion channel complex that is crucial in the regulation of firing frequency and synaptic plasticity [88,90,92,93]. Interaction of DPP6 with the permeation and gating modules of the KV4 channels facilitated inactivation [94,95].

The neuronal membrane glycoprotein M6-a (M6a) is a 278-amino acid transmembrane glycoprotein with four transmembrane domains with one intracellular and two extracellular loops and the N- and C-termini located in the cytoplasm [96]. It is the only member of the proteolipid protein family of tetraspan proteins to be expressed exclusively in the central nervous system [97]. M6a expression was particularly strong in unmyelinated axonal fibers such as cerebellar parallel fibers [98]. M6a has been suspected to play a role in the formation of nerve cell processes since in cultured cerebellar neurons treated with monoclonal M6a antibody, neurite formation was severely impaired [99]. Strong labeling for M6a was observed in the cerebellar molecular layer corresponding to heavily stained axon terminals originating from granule cells. As observed by immune electron microscopy M6a was present only on the cytoplasmic side of the presynaptic membrane and on the membrane of synaptic vesicles [100]. M6a has been allocated to the leading edge of growth cones in cultured cerebellar neurons [101] and in lipid rafts in membrane microdomains where it induced filopodia formation [102].

Proline-rich transmembrane protein 2 (PRRT2) is a largely uncharacterized protein. It is expressed in the brain and has been demonstrated to interact with SNAP-25, a component of the molecular machinery involved in the release of neurotransmitters at the presynaptic plasma membrane [103]. PRRT2 has been described to play a role in Paroxysmal Kinesigenic Dyskenia, a dominant movement disorder [104], as well as in other neurological disorders [105].

The function of *N*-myc downstream-regulated gene 2 (NDRG2) protein is unknown, however it is believed to be involved in cell growth events [106]. Using subtractive cloning technology it was shown that the A/B-hydrolase fold protein gene NDRG2 (NDRG family member 2) was upregulated at both the

RNA and protein levels in Alzheimer´s disease brains. Expression of NDRG2 in affected brains was revealed in cortical pyramidal neurons, senile plaques and cellular processes of dystrophic neurons [107].

The inositol 1,4,5-trisphosphate receptor type 1, also named Purkinje cell protein 1, is a ubiquitous 250 kDa major phosphorylated glycoprotein especially abundant in cerebellar Purkinje cells at the plasma membrane [108–111]. Intracranial injection of 14C-leucine, revealed that inositol 1,4,5-trisphosphate receptor type 1 was one of the dominant proteins in the cerebellum. In Purkinje cell deleted mutant mice no inositol 1,4,5-trisphosphate receptor type 1 was present [111].

Phosphatidylethanolamine-binding protein 1 (PEBP-1) is largely undescribed. It has been involved in the function of presynaptic cholinergic neurons of the central nervous system in respect of learning and memory [71].

References

1. Harashima, S.; Wang, Y.; Horiuchi, T.; Seino, Y.; Inagaki, N. Purkinje cell protein 4 positively regulates neurite outgrowth and neurotransmitter release. *J. Neurosci. Res.* **2011**, *89*, 1519–1530.
2. Sangameswaran, L.; Hempstead, J.; Morgan, J.I. Molecular cloning of a neuron-specific transcript and its regulation during normal and aberrant cerebellar development. *Proc. Natl. Acad. Sci. USA* **1989**, *86*, 5651–5655.
3. Ziai, M.R.; Sangameswaran, L.; Hempstead, J.L.; Danho, W.; Morgan, J.I. An immunochemical analysis of the distribution of a brain-specific polypeptide, pep-19. *J. Neurochem.* **1988**, *51*, 1771–1776.
4. Simons, S.B.; Escobedo, Y.; Yasuda, R.; Dudek, S.M. Regional differences in hippocampal calcium handling provide a cellular mechanism for limiting plasticity. *Proc. Natl. Acad. Sci. USA* **2009**, *106*, 14080–14084.
5. Slemmon, J.R.; Morgan, J.I.; Fullerton, S.M.; Danho, W.; Hilbush, B.S.; Wengenack, T.M. Camstatins are peptide antagonists of calmodulin based upon a conserved structural motif in pep-19, neurogranin, and neuromodulin. *J. Biol. Chem.* **1996**, *271*, 15911–15917.
6. Maurya, D.K.; Sundaram, C.S.; Bhargava, P. Proteome profile of the mature rat olfactory bulb. *Proteomics* **2009**, *9*, 2593–2599.
7. Ramakers, G.J.; Verhaagen, J.; Oestreicher, A.B.; Margolis, F.L.; van Bergen en Henegouwen, P.M.; Gispen, W.H. Immunolocalization of b-50 (gap-43) in the mouse olfactory bulb: Predominant presence in preterminal axons. *J. Neurocytol.* **1992**, *21*, 853–869.
8. Skene, J.H.; Willard, M. Changes in axonally transported proteins during axon regeneration in toad retinal ganglion cells. *J. Cell Biol.* **1981**, *89*, 86–95.
9. Skene, J.H.; Willard, M. Electrophoretic analysis of axonally transported proteins in toad retinal ganglion cells. *J. Neurochem.* **1981**, *37*, 79–87.
10. Dekker, L.V.; De Graan, P.N.; Versteeg, D.H.; Oestreicher, A.B.; Gispen, W.H. Phosphorylation of b-50 (gap43) is correlated with neurotransmitter release in rat hippocampal slices. *J. Neurochem.* **1989**, *52*, 24–30.
11. Lovinger, D.M.; Akers, R.F.; Nelson, R.B.; Barnes, C.A.; McNaughton, B.L.; Routtenberg, A. A selective increase in phosporylation of protein f1, a protein kinase c substrate, directly related to three day growth of long term synaptic enhancement. *Brain Res.* **1985**, *343*, 137–143.

12. Camoletto, P.; Peretto, P.; Bonfanti, L.; Manceau, V.; Sobel, A.; Fasolo, A. The cytosolic phosphoprotein stathmin is expressed in the olfactory system of the adult rat. *Neuroreport* **1997**, *8*, 2825–2829.
13. Grenningloh, G.; Soehrman, S.; Bondallaz, P.; Ruchti, E.; Cadas, H. Role of the microtubule destabilizing proteins scg10 and stathmin in neuronal growth. *J. Neurobiol.* **2004**, *58*, 60–69.
14. Camoletto, P.; Colesanti, A.; Ozon, S.; Sobel, A.; Fasolo, A. Expression of stathmin and scg10 proteins in the olfactory neurogenesis during development and after lesion in the adulthood. *Brain Res. Bull.* **2001**, *54*, 19–28.
15. Pellier-Monnin, V.; Astic, L.; Bichet, S.; Riederer, B.M.; Grenningloh, G. Expression of scg10 and stathmin proteins in the rat olfactory system during development and axonal regeneration. *J. Comp. Neurol.* **2001**, *433*, 239–254.
16. Jin, K.; Mao, X.O.; Cottrell, B.; Schilling, B.; Xie, L.; Row, R.H.; Sun, Y.; Peel, A.; Childs, J.; Gendeh, G.; *et al.* Proteomic and immunochemical characterization of a role for stathmin in adult neurogenesis. *FASEB J. Off. Publ. Fed. Am. Soc. Exp. Biol.* **2004**, *18*, 287–299.
17. Curmi, P.A.; Andersen, S.S.; Lachkar, S.; Gavet, O.; Karsenti, E.; Knossow, M.; Sobel, A. The stathmin/tubulin interaction *in vitro*. *J. Biol. Chem.* **1997**, *272*, 25029–25036.
18. Amat, J.A.; Fields, K.L.; Schubart, U.K. Distribution of phosphoprotein p19 in rat brain during ontogeny: Stage-specific expression in neurons and glia. *Brain Res. Dev. Brain Res.* **1991**, *60*, 205–218.
19. Margolis, F.L. A brain protein unique to the olfactory bulb. *Proc. Natl. Acad. Sci. USA* **1972**, *69*, 1221–1224.
20. Graziadei, G.A.; Stanley, R.S.; Graziadei, P.P. The olfactory marker protein in the olfactory system of the mouse during development. *Neuroscience* **1980**, *5*, 1239–1252.
21. Kasowski, H.J.; Kim, H.; Greer, C.A. Compartmental organization of the olfactory bulb glomerulus. *J. Comp. Neurol.* **1999**, *407*, 261–274.
22. Lee, A.C.; He, J.; Ma, M. Olfactory marker protein is critical for functional maturation of olfactory sensory neurons and development of mother preference. *J. Neurosci. Off. J. Soc. Neurosci.* **2011**, *31*, 2974–2982.
23. Philpot, B.D.; Lim, J.H.; Brunjes, P.C. Activity-dependent regulation of calcium-binding proteins in the developing rat olfactory bulb. *J. Comp. Neurol.* **1997**, *387*, 12–26.
24. Qin, Z.P.; Ye, S.M.; Du, J.Z.; Shen, G.Y. Postnatal developmental expression of calbindin, calretinin and parvalbumin in mouse main olfactory bulb. *Acta Biochim. Biophys. Sinica* **2005**, *37*, 276–282.
25. Bastianelli, E.; Pochet, R. Calmodulin, calbindin-d28k, calretinin and neurocalcin in rat olfactory bulb during postnatal development. *Brain Res. Dev. Brain Res.* **1995**, *87*, 224–227.
26. Rogers, J.H. Immunohistochemical markers in rat brain: Colocalization of calretinin and calbindin-d28k with tyrosine hydroxylase. *Brain Res.* **1992**, *587*, 203–210.
27. Wouterlood, F.G.; Hrtig, W. Calretinin-immunoreactivity in mitral cells of the rat olfactory bulb. *Brain Res.* **1995**, *682*, 93–100.
28. Bedard, A.; Parent, A. Evidence of newly generated neurons in the human olfactory bulb. *Brain Res. Dev. Brain Res.* **2004**, *151*, 159–168.

29. Winner, B.; Cooper-Kuhn, C.M.; Aigner, R.; Winkler, J.; Kuhn, H.G. Long-term survival and cell death of newly generated neurons in the adult rat olfactory bulb. *Eur. J. Neurosci.* **2002**, *16*, 1681–1689.
30. Hoogland, P.V.; Huisman, E. Tyrosine hydroxylase immunoreactive structures in the aged human olfactory bulb and olfactory peduncle. *J. Chem. Neuroanat.* **1999**, *17*, 153–161.
31. Celio, M.R. Calbindin d-28k and parvalbumin in the rat nervous system. *Neuroscience* **1990**, *35*, 375–475.
32. Heizmann, C.W. Parvalbumin, an intracellular calcium-binding protein; distribution, properties and possible roles in mammalian cells. *Experientia* **1984**, *40*, 910–921.
33. Basile, M.; Lin, R.; Kabbani, N.; Karpa, K.; Kilimann, M.; Simpson, I.; Kester, M. Paralemmin interacts with d3 dopamine receptors: Implications for membrane localization and camp signaling. *Arch. Biochem. Biophys.* **2006**, *446*, 60–68.
34. Jones, D.T.; Reed, R.R. Golf: An olfactory neuron specific-g protein involved in odorant signal transduction. *Science* **1989**, *244*, 790–795.
35. Halpern, M.; Shapiro, L.S.; Jia, C. Heterogeneity in the accessory olfactory system. *Chem. Senses* **1998**, *23*, 477-481.
36. Jia, C.; Halpern, M. Subclasses of vomeronasal receptor neurons: Differential expression of G proteins (Gi alpha 2 and G(o alpha)) and segregated projections to the accessory olfactory bulb. *Brain Res.* **1996**, *719*, 117–128.
37. Zhu, P.P.; Patterson, A.; Lavoie, B.; Stadler, J.; Shoeb, M.; Patel, R.; Blackstone, C. Cellular localization, oligomerization, and membrane association of the hereditary spastic paraplegia 3a (spg3a) protein atlastin. *J. Biolog. Chem.* **2003**, *278*, 49063–49071.
38. Zhu, P.P.; Soderblom, C.; Tao-Cheng, J.H.; Stadler, J.; Blackstone, C. Spg3a protein atlastin-1 is enriched in growth cones and promotes axon elongation during neuronal development. *Hum. Mol. Genet.* **2006**, *15*, 1343–1353.
39. Murai, K.K.; Misner, D.; Ranscht, B. Contactin supports synaptic plasticity associated with hippocampal long-term depression but not potentiation. *Curr. Biol. CB* **2002**, *12*, 181–190.
40. Shimazaki, K.; Hosoya, H.; Takeda, Y.; Kobayashi, S.; Watanabe, K. Age-related decline of f3/contactin in rat hippocampus. *Neurosci. Lett.* **1998**, *245*, 117–120.
41. Puzzo, D.; Bizzoca, A.; Privitera, L.; Furnari, D.; Giunta, S.; Girolamo, F.; Pinto, M.; Gennarini, G.; Palmeri, A. F3/contactin promotes hippocampal neurogenesis, synaptic plasticity, and memory in adult mice. *Hippocampus* **2013**, *23*, 1367–1382.
42. Wang, H.; Nong, Y.; Bazan, F.; Greengard, P.; Flajolet, M. Norbin: A promising central nervous system regulator. *Commun. Integr. Biol.* **2010**, *3*, 487–490.
43. Wang, H.; Westin, L.; Nong, Y.; Birnbaum, S.; Bendor, J.; Brismar, H.; Nestler, E.; Aperia, A.; Flajolet, M.; Greengard, P. Norbin is an endogenous regulator of metabotropic glutamate receptor 5 signaling. *Science* **2009**, *326*, 1554–1557.
44. Shinozaki, K.; Kume, H.; Kuzume, H.; Obata, K.; Maruyama, K. Norbin, a neurite-outgrowth-related protein, is a cytosolic protein localized in the somatodendritic region of neurons and distributed prominently in dendritic outgrowth in purkinje cells. *Brain Res. Mol. Brain Res.* **1999**, *71*, 364–368.

45. Dateki, M.; Horii, T.; Kasuya, Y.; Mochizuki, R.; Nagao, Y.; Ishida, J.; Sugiyama, F.; Tanimoto, K.; Yagami, K.; Imai, H.; *et al.* Neurochondrin negatively regulates camkii phosphorylation, and nervous system-specific gene disruption results in epileptic seizure. *J. Biol. Chem.* **2005**, *280*, 20503–20508.
46. Price, N.E.; Wadzinski, B.; Mumby, M.C. An anchoring factor targets protein phosphatase 2a to brain microtubules. *Brain Res. Mol. Brain Res.* **1999**, *73*, 68–77.
47. Thiels, E.; Norman, E.D.; Barrionuevo, G.; Klann, E. Transient and persistent increases in protein phosphatase activity during long-term depression in the adult hippocampus *in vivo*. *Neuroscience* **1998**, *86*, 1023–1029.
48. Mulkey, R.M.; Herron, C.E.; Malenka, R.C. An essential role for protein phosphatases in hippocampal long-term depression. *Science* **1993**, *261*, 1051–1055.
49. Norman, E.D.; Thiels, E.; Barrionuevo, G.; Klann, E. Long-term depression in the hippocampus *in vivo* is associated with protein phosphatase-dependent alterations in extracellular signal-regulated kinase. *J. Neurochem.* **2000**, *74*, 192–198.
50. Liu, R.; Zhou, X.W.; Tanila, H.; Bjorkdahl, C.; Wang, J.Z.; Guan, Z.Z.; Cao, Y.; Gustafsson, J.A.; Winblad, B.; Pei, J.J. Phosphorylated pp2a (tyrosine 307) is associated with alzheimer neurofibrillary pathology. *J. Cell. Mol. Med.* **2008**, *12*, 241–257.
51. Vogelsberg-Ragaglia, V.; Schuck, T.; Trojanowski, J.Q.; Lee, V.M. Pp2a mrna expression is quantitatively decreased in alzheimer's disease hippocampus. *Exp. Neurol.* **2001**, *168*, 402–412.
52. Rahman, A.; Khan, K.M.; Al-Khaledi, G.; Khan, I.; Al-Shemary, T. Over activation of hippocampal serine/threonine protein phosphatases pp1 and pp2a is involved in lead-induced deficits in learning and memory in young rats. *Neurotoxicology* **2012**, *33*, 370–383.
53. Kartmann, B.; Roth, D. Novel roles for mammalian septins: From vesicle trafficking to oncogenesis. *J. Cell Sci.* **2001**, *114*, 839–844.
54. Barral, Y.; Kinoshita, M. Structural insights shed light onto septin assemblies and function. *Curr. Opin. Cell Biol.* **2008**, *20*, 12–18.
55. Joo, E.; Tsang, C.W.; Trimble, W.S. Septins: Traffic control at the cytokinesis intersection. *Traffic* **2005**, *6*, 626–634.
56. Weirich, C.S.; Erzberger, J.P.; Barral, Y. The septin family of gtpases: Architecture and dynamics. *Nature reviews. Mol. Cell Biol.* **2008**, *9*, 478–489.
57. Kinoshita, A.; Noda, M.; Kinoshita, M. Differential localization of septins in the mouse brain. *J. Comp. Neurol.* **2000**, *428*, 223–239.
58. Fujishima, K.; Kiyonari, H.; Kurisu, J.; Hirano, T.; Kengaku, M. Targeted disruption of sept3, a heteromeric assembly partner of sept5 and sept7 in axons, has no effect on developing cns neurons. *J. Neurochem.* **2007**, *102*, 77–92.
59. Ihara, M.; Yamasaki, N.; Hagiwara, A.; Tanigaki, A.; Kitano, A.; Hikawa, R.; Tomimoto, H.; Noda, M.; Takanashi, M.; Mori, H.; *et al.* Sept4, a component of presynaptic scaffold and lewy bodies, is required for the suppression of alpha-synuclein neurotoxicity. *Neuron* **2007**, *53*, 519–533.
60. Son, J.H.; Kawamata, H.; Yoo, M.S.; Kim, D.J.; Lee, Y.K.; Kim, S.; Dawson, T.M.; Zhang, H.; Sulzer, D.; Yang, L.; *et al.* Neurotoxicity and behavioral deficits associated with septin 5 accumulation in dopaminergic neurons. *J. Neurochem.* **2005**, *94*, 1040–1053.

61. Sudo, K.; Ito, H.; Iwamoto, I.; Morishita, R.; Asano, T.; Nagata, K. Sept9 sequence alternations causing hereditary neuralgic amyotrophy are associated with altered interactions with sept4/sept11 and resistance to rho/rhotekin-signaling. *Hum. Mutat.* **2007**, *28*, 1005–1013.
62. Blondeau, F.; Ritter, B.; Allaire, P.D.; Wasiak, S.; Girard, M.; Hussain, N.K.; Angers, A.; Legendre-Guillemin, V.; Roy, L.; Boismenu, D.; *et al.* Tandem ms analysis of brain clathrin-coated vesicles reveals their critical involvement in synaptic vesicle recycling. *Proc. Natl. Acad. Sci. USA* **2004**, *101*, 3833–3838.
63. Burre, J.; Beckhaus, T.; Schagger, H.; Corvey, C.; Hofmann, S.; Karas, M.; Zimmermann, H.; Volknandt, W. Analysis of the synaptic vesicle proteome using three gel-based protein separation techniques. *Proteomics* **2006**, *6*, 6250–6262.
64. Takamori, S.; Holt, M.; Stenius, K.; Lemke, E.A.; Gronborg, M.; Riedel, D.; Urlaub, H.; Schenck, S.; Brugger, B.; Ringler, P.; *et al.* Molecular anatomy of a trafficking organelle. *Cell* **2006**, *127*, 831–846.
65. Xue, J.; Tsang, C.W.; Gai, W.P.; Malladi, C.S.; Trimble, W.S.; Rostas, J.A.; Robinson, P.J. Septin 3 (g-septin) is a developmentally regulated phosphoprotein enriched in presynaptic nerve terminals. *J. Neurochem.* **2004**, *91*, 579–590.
66. Morciano, M.; Beckhaus, T.; Karas, M.; Zimmermann, H.; Volknandt, W. The proteome of the presynaptic active zone: From docked synaptic vesicles to adhesion molecules and maxi-channels. *J. Neurochem.* **2009**, *108*, 662–675.
67. Weingarten, J.; Lassek, M.; Mueller, B.F.; Rohmer, M.; Lunger, I.; Baeumlisberger, D.; Dudek, S.; Gogesch, P.; Karas, M.; Volknandt, W. The proteome of the presynaptic active zone from mouse brain. *Mol. Cell. Neurosci.* **2014**, *59C*, 106–118.
68. Tsang, C.W.; Estey, M.P.; DiCiccio, J.E.; Xie, H.; Patterson, D.; Trimble, W.S. Characterization of presynaptic septin complexes in mammalian hippocampal neurons. *Biol. Chem.* **2011**, *392*, 739–749.
69. Fountoulakis, M.; Tsangaris, G.T.; Maris, A.; Lubec, G. The rat brain hippocampus proteome. *J. Chromatogr. B* **2005**, *819*, 115–129.
70. Castegna, A.; Aksenov, M.; Thongboonkerd, V.; Klein, J.B.; Pierce, W.M.; Booze, R.; Markesbery, W.R.; Butterfield, D.A. Proteomic identification of oxidatively modified proteins in alzheimer's disease brain. Part II: Dihydropyrimidinase-related protein 2, α-enolase and heat shock cognate 71. *J. Neurochem.* **2002**, *82*, 1524–1532.
71. Piubelli, C.; Carboni, L.; Becchi, S.; Mathe, A.A.; Domenici, E. Regulation of cytoskeleton machinery, neurogenesis and energy metabolism pathways in a rat gene-environment model of depression revealed by proteomic analysis. *Neuroscience* **2011**, *176*, 349–380.
72. Piubelli, C.; Gruber, S.; El Khoury, A.; Mathe, A.A.; Domenici, E.; Carboni, L. Nortriptyline influences protein pathways involved in carbohydrate metabolism and actin-related processes in a rat gene-environment model of depression. *Eur. Neuropsychopharmacol. J. Eur. Coll. Neuropsychopharmacol.* **2011**, *21*, 545–562.
73. Reed, T.T.; Pierce, W.M.; Markesbery, W.R.; Butterfield, D.A. Proteomic identification of hne-bound proteins in early alzheimer disease: Insights into the role of lipid peroxidation in the progression of ad. *Brain Res.* **2009**, *1274*, 66–76.

74. Imperlini, E.; Orru, S.; Corbo, C.; Daniele, A.; Salvatore, F. Altered brain protein expression profiles are associated with molecular neurological dysfunction in the pku mouse model. *J. Neurochem.* **2014**, *129*, 1002–1012.
75. Kobeissy, F.H.; Warren, M.W.; Ottens, A.K.; Sadasivan, S.; Zhang, Z.; Gold, M.S.; Wang, K.K. Psychoproteomic analysis of rat cortex following acute methamphetamine exposure. *J. Proteome Res.* **2008**, *7*, 1971–1983.
76. Rohe, M.; Nebrich, G.; Klein, O.; Mao, L.; Zabel, C.; Klose, J.; Hartl, D. Kainate promotes alterations in neuronal RNA splicing machinery. *J. Proteome Res.* **2011**, *10*, 1459–1467.
77. Sacksteder, C.A.; Qian, W.J.; Knyushko, T.V.; Wang, H.; Chin, M.H.; Lacan, G.; Melega, W.P.; Camp, D.G., 2nd; Smith, R.D.; Smith, D.J.; *et al.* Endogenously nitrated proteins in mouse brain: Links to neurodegenerative disease. *Biochemistry* **2006**, *45*, 8009–8022.
78. Dhaunchak, A.S.; Huang, J.K.; De Faria Junior, O.; Roth, A.D.; Pedraza, L.; Antel, J.P.; Bar-Or, A.; Colman, D.R. A proteome map of axoglial specializations isolated and purified from human central nervous system. *Glia* **2010**, *58*, 1949–1960.
79. Patil, S.S.; Li, K.; Heo, S.; Hoger, H.; Lubec, G. Proteins linked to spatial memory formation of cd1 mice in the multiple t-maze. *Hippocampus* **2012**, *22*, 1075–1086.
80. Manadas, B.; Santos, A.R.; Szabadfi, K.; Gomes, J.R.; Garbis, S.D.; Fountoulakis, M.; Duarte, C.B. Bdnf-induced changes in the expression of the translation machinery in hippocampal neurons: Protein levels and dendritic mrna. *J. Proteome Res.* **2009**, *8*, 4536–4552.
81. Schwaller, B.; Meyer, M.; Schiffmann, S. 'New' functions for 'old' proteins: The role of the calcium-binding proteins calbindin d-28k, calretinin and parvalbumin, in cerebellar physiology. Studies with knockout mice. *Cerebellum* **2002**, *1*, 241–258.
82. Enderlin, S.; Norman, A.W.; Celio, M.R. Ontogeny of the calcium binding protein calbindin d-28k in the rat nervous system. *Anat. Embryol.* **1987**, *177*, 15–28.
83. Garcia-Segura, L.M.; Baetens, D.; Roth, J.; Norman, A.W.; Orci, L. Immunohistochemical mapping of calcium-binding protein immunoreactivity in the rat central nervous system. *Brain Res.* **1984**, *296*, 75–86.
84. Iacopino, A.M.; Rhoten, W.B.; Christakos, S. Calcium binding protein (calbindin-d28k) gene expression in the developing and aging mouse cerebellum. *Brain Res. Mol. Brain Res.* **1990**, *8*, 283–290.
85. Baimbridge, K.G.; Miller, J.J.; Parkes, C.O. Calcium-binding protein distribution in the rat brain. *Brain Res.* **1982**, *239*, 519–525.
86. Barski, J.J.; Hartmann, J.; Rose, C.R.; Hoebeek, F.; Morl, K.; Noll-Hussong, M.; de Zeeuw, C.I.; Konnerth, A.; Meyer, M. Calbindin in cerebellar purkinje cells is a critical determinant of the precision of motor coordination. *J. Neurosci.: Off. J. Soc. Neurosci.* **2003**, *23*, 3469–3477.
87. Airaksinen, M.S.; Eilers, J.; Garaschuk, O.; Thoenen, H.; Konnerth, A.; Meyer, M. Ataxia and altered dendritic calcium signaling in mice carrying a targeted null mutation of the calbindin d28k gene. *Proc. Natl. Acad. Sci. USA* **1997**, *94*, 1488–1493.
88. Clark, B.D.; Kwon, E.; Maffie, J.; Jeong, H.Y.; Nadal, M.; Strop, P.; Rudy, B. Dpp6 localization in brain supports function as a kv4 channel associated protein. *Front. Mol. Neurosci.* **2008**, *1*, 8.

89. Kin, Y.; Misumi, Y.; Ikehara, Y. Biosynthesis and characterization of the brain-specific membrane protein dppx, a dipeptidyl peptidase iv-related protein. *J. Biochem.* **2001**, *129*, 289–295.
90. Nadin, B.M.; Pfaffinger, P.J. A new task for dipeptidyl peptidase-like protein 6. *PLoS ONE* **2013**, *8*, e60831.
91. Nadin, B.M.; Pfaffinger, P.J. Dipeptidyl peptidase-like protein 6 is required for normal electrophysiological properties of cerebellar granule cells. *J. Neurosci. Off. J. Soc. Neurosci.* **2010**, *30*, 8551–8565.
92. Attali, B. The somato-dendritic a-type k channel complex: A menage a trois. *Front. Neurosci.* **2009**, *3*, 158–159.
93. Nadal, M.S.; Ozaita, A.; Amarillo, Y.; Vega-Saenz de Miera, E.; Ma, Y.; Mo, W.; Goldberg, E.M.; Misumi, Y.; Ikehara, Y.; Neubert, T.A.; *et al.* The cd26-related dipeptidyl aminopeptidase-like protein dppx is a critical component of neuronal a-type k+ channels. *Neuron* **2003**, *37*, 449–461.
94. Kaulin, Y.A.; De Santiago-Castillo, J.A.; Rocha, C.A.; Nadal, M.S.; Rudy, B.; Covarrubias, M. The dipeptidyl-peptidase-like protein dpp6 determines the unitary conductance of neuronal kv4.2 channels. *J. Neurosci. Off. J. Soc. Neurosci.* **2009**, *29*, 3242–3251.
95. Ren, X.; Hayashi, Y.; Yoshimura, N.; Takimoto, K. Transmembrane interaction mediates complex formation between peptidase homologues and kv4 channels. *Mol. Cell. Neurosci.* **2005**, *29*, 320–332.
96. Brocco, M.A.; Fernandez, M.E.; Frasch, A.C. Filopodial protrusions induced by glycoprotein m6a exhibit high motility and aids synapse formation. *Eur. J. Neurosci.* **2010**, *31*, 195–202.
97. Yan, Y.; Narayanan, V.; Lagenaur, C. Expression of members of the proteolipid protein gene family in the developing murine central nervous system. *J. Comp. Neurol.* **1996**, *370*, 465–478.
98. Cooper, B.; Werner, H.B.; Flugge, G. Glycoprotein m6a is present in glutamatergic axons in adult rat forebrain and cerebellum. *Brain Res.* **2008**, *1197*, 1–12.
99. Lagenaur, C.; Kunemund, V.; Fischer, G.; Fushiki, S.; Schachner, M. Monoclonal m6 antibody interferes with neurite extension of cultured neurons. *J. Neurobiol.* **1992**, *23*, 71–88.
100. Roussel, G.; Trifilieff, E.; Lagenaur, C.; Nussbaum, J.L. Immunoelectron microscopic localization of the m6a antigen in rat brain. *J. Neurocytol.* **1998**, *27*, 695–703.
101. Mukobata, S.; Hibino, T.; Sugiyama, A.; Urano, Y.; Inatomi, A.; Kanai, Y.; Endo, H.; Tashiro, F. M6a acts as a nerve growth factor-gated Ca^{2+} channel in neuronal differentiation. *Biochem. Biophys. Res. Commun.* **2002**, *297*, 722–728.
102. Scorticati, C.; Formoso, K.; Frasch, A.C. Neuronal glycoprotein M6A induces filopodia formation via association with cholesterol-rich lipid rafts. *J. Neurochem.* **2011**, *119*, 521–531.
103. Heron, S.E.; Dibbens, L.M. Role of prrt2 in common paroxysmal neurological disorders: A gene with remarkable pleiotropy. *J. Med. Genet.* **2013**, *50*, 133–139.
104. Liu, X.R.; Wu, M.; He, N.; Meng, H.; Wen, L.; Wang, J.L.; Zhang, M.P.; Li, W.B.; Mao, X.; Qin, J.M.; *et al.* Novel PRRT2 mutations in paroxysmal dyskinesia patients with variant inheritance and phenotypes. *Genes Brain Behav.* **2013**, *12*, 234–240.

105. Heron, S.E.; Grinton, B.E.; Kivity, S.; Afawi, Z.; Zuberi, S.M.; Hughes, J.N.; Pridmore, C.; Hodgson, B.L.; Iona, X.; Sadleir, L.G.; *et al.* Prrt2 mutations cause benign familial infantile epilepsy and infantile convulsions with choreoathetosis syndrome. *Am. J. Hum. Genet.* **2012**, *90*, 152–160.
106. Hu, X.L.; Liu, X.P.; Deng, Y.C.; Lin, S.X.; Wu, L.; Zhang, J.; Wang, L.F.; Wang, X.B.; Li, X.; Shen, L.; *et al.* Expression analysis of the NDRG2 gene in mouse embryonic and adult tissues. *Cell Tissue Res.* **2006**, *325*, 67–76.
107. Mitchelmore, C.; Buchmann-Moller, S.; Rask, L.; West, M.J.; Troncoso, J.C.; Jensen, N.A. Ndrg2: A novel alzheimer's disease associated protein. *Neurobiol. Dis.* **2004**, *16*, 48–58.
108. Maeda, N.; Niinobe, M.; Inoue, Y.; Mikoshiba, K. Developmental expression and intracellular location of p400 protein characteristic of purkinje cells in the mouse cerebellum. *Dev. Biol.* **1989**, *133*, 67–76.
109. Maeda, N.; Niinobe, M.; Mikoshiba, K. A cerebellar purkinje cell marker p400 protein is an inositol 1,4,5-trisphosphate (Insp3) receptor protein. Purification and characterization of INSP3 receptor complex. *EMBO J.* **1990**, *9*, 6–67.
110. Mikoshiba, K.; Changeux, J.P. Morphological and biochemical studies on isolated molecular and granular layers from bovine cerebellum. *Brain Res.* **1978**, *142*, 487–504.
111. Mikoshiba, K.; Okano, H.; Tsukada, Y. P400 protein characteristic to purkinje cells and related proteins in cerebella from neuropathological mutant mice: Autoradiographic study by 14c-leucine and phosphorylation. *Dev. Neurosci.* **1985**, *7*, 179–187.
